# Joining of Silicon Particle-Reinforced Aluminum Matrix Composites to Kovar Alloys Using Active Melt-Spun Ribbons in Vacuum Conditions

**DOI:** 10.3390/ma13132965

**Published:** 2020-07-02

**Authors:** Zeng Gao, Xianli Ba, Huanyu Yang, Congxin Yin, Shanguang Liu, Jitai Niu, Josip Brnic

**Affiliations:** 1 School of Materials Science and Engineering, Henan Polytechnic University, Jiaozuo 454003, China; baxianli112233@163.com (X.B.); yanghuanyuyhy@163.com (H.Y.); Ycx7469@163.com (C.Y.); niujitai@163.com (J.N.); 2 Beijing Institute of Aeronautical Materials, Beijing 100095, China; liusg621@126.com; 3 School of Materials Science and Engineering, Harbin Institute of Technology, Harbin 150001, China; 4 Faculty of Engineering, University of Rijeka, 51000 Rijeka, Croatia; brnic@riteh.hr

**Keywords:** Si_p_/Al MMCs, Kovar, vacuum brazing, micro nano ribbon, microstructure

## Abstract

The vacuum brazing of dissimilar electronic packaging materials has been investigated. In this research, this applies silicon particle-reinforced aluminum matrix composites (Si_p_/Al MMCs) to Kovar alloys. Active melt-spun ribbons were employed as brazing filler metals under different joining temperatures and times. The results showed that the maximum joint shear strength of 96.62 MPa was achieved when the joint was made using Al-7.5Si-23.0Cu-2.0Ni-1.0Ti as the brazing filler metal at 580 °C for 30 min. X-ray diffraction (XRD) analysis of the joint indicated that the main phases were composed of Al, Si and intermetallics, including CuAl, TiFeSi, TiNiSi and Al_3_Ti. When the brazing temperature ranged from 570 °C to 590 °C, the leakage rate of joints remained at 10^−8^ Pa·m^3^/s or better. When the joint was made using Al-7.5Si-23.0Cu-2.0Ni-2.5Ti as the brazing filler metal at 580 °C for 30 min, the higher level of Ti content in the brazing filler metal resulted in the formation of a flake-like Ti(AlSi)_3_ intermetallic phase with an average size of 7 µm at the interface between the brazing seam and Si_p_/Al MMCs. The joint fracture was generally in the form of quasi-cleavage fracture, which primarily occurred at the interface between the filler metal and the Si_p_/Al MMCs. The micro-crack propagated not only Ti(AlSi)_3_, but also the Si particles in the substrate.

## 1. Introduction

With the rapid development of the aerospace industry, the electronic products in aerobat are tending towards miniaturization and high-power density, aiming to reduce the component weight. Consequently, this leads to increased requirements for electronic packaging materials, such as light weight, low coefficient of thermal expansion and high thermal conductivity [1,2,3,4]. In addition to this, the performance of conventional packaging materials can no longer meet the requirements of microelectronic technology development in the aerospace and automotive industries [5,6]. Electronic packaging components are subjected to random loading and moisture erosion in service. Therefore, the appropriate mechanical properties and gas tightness of the electronic packaging components manufactured by the joining process are requested [7,8,9,10].

Among the numerous advanced electronic packaging materials, silicon particle-reinforced aluminum matrix composites (Si_p_/Al MMCs) possess comprehensive performances that make them more promising and attractive for their application in the aerospace field instead of the conventional heavy packaging materials. Si_p_/Al MMCs (Si content from 27–70 wt.%) can be manufactured using spray casting technology by improving the silicon supersaturation in the aluminum matrix. The advanced material of Si_p_/Al MMCs has characteristics such as a low coefficient of thermal expansion, low density, high thermal conductivity, good thermo-mechanical stability and is compatible with microelectronic assembly technology, etc. [11,12,13,14]. Due to the homogeneous distribution of Si particles in composites, components made from Si_p_/Al MMCs are nearly isotropic and readily formable [15,16]. Moreover, this material can be customized to meet specific requirements such as different coefficients of thermal expansion and thermal conductivity via changes in the Si content in the composites, making them especially attractive for aerospace engineering and the automotive industry, in addition to their electronic packaging applications [17,18]. Kovar alloy, also known as controlled expansion alloy or sealing alloy, since it has a similar coefficient of thermal expansion to glass and ceramics, so as to achieve a matching sealing requirement with the sealed materials [19,20,21]. As a conventional packaging material, Kovar alloy has been used at various electronic components for several decades, proving its low coefficient of thermal expansion, good machinability and thermostability, etc. [20,22]. For further applications of advanced electronic packaging materials, the joining of dissimilar packaging materials is inevitable due to the requirements of component design and manufacturing. Sometimes, the joining technology may become the key issue limiting the use of these advanced composites. As Kovar alloys and Si_p_/Al MMCs have large differences in several areas such as their thermal, physical and mechanical properties, the joining process is challenging because thermal stress easily forms at the joints, causing micro-cracking, leakage and even joint breakage. However, very few studies in the literature can be found that deal with the joining problem of Si_p_/Al MMCs with Kovar.

This work mainly describes the joining of Si_p_/Al MMCs to Kovar alloys using active melt-spun ribbon as the filler metal in vacuum conditions. The microstructural characteristics and thermodynamic properties of brazing filler metal prepared by melt spinning technology were investigated primarily. Different brazing temperatures, brazing times and filler metals with different active Ti additions were considered in this study to investigate the effects of Ti addition on the brazing filler metal and the process parameters on the joint microstructural evolution and fracture morphology, as well as on the joint properties, such as shear strength and gas tightness. This study provides a new approach for joining the dissimilar electronic packaging materials of Si_p_/Al MMCs and Kovar used in the aerospace industry.

## 2. Materials and Methods

Commercially available Kovar alloys and Si_p_/Al MMCs (Harvest Technology Development Company, Ltd., Changsha, China) were used in this investigation. The nominal composition of the conventional electronic packaging material Kovar alloy 4J29 used in this study consists of Fe54-Co17-Ni29. Kovar represented single-phase austenite in the temperature range of −70 °C to 1000 °C, which exhibited high thermostability [23]. The chemical composition of the Si_p_/Al MMCs is presented in Table 1.

**Figure 1:**
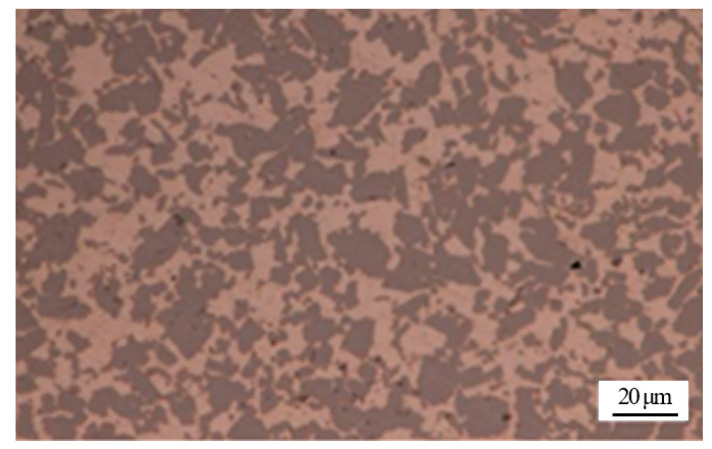
Microstructure of 50 wt.% Si_p_/Al MMCs manufactured by spray casting technique.

As a novel packaging material, 50 wt.% Si_p_/Al MMCs were produced using spray casting technique, in which the liquid metal droplets with sizes between 10 µm and 500 µm were continuously sprayed on deposition plates through an atomization device. On account of the atomizing and rapid cooling effects during the manufacturing process, the supersaturated second-phase silicon particle in the Al-Si system can be micronized and uniformly distributed. Figure 1 exhibits the microstructure of 50 wt.% Si_p_/Al MMCs. As can be seen, the silicon particles with a size not exceeding 18 µm were distributed in the base aluminum alloy quite uniformly. As known from the Al-Si binary phase diagram, Si content in hypereutectic Al-Si alloys exceeded 12.6% (in wt.%). After a traditional solidification process, the microstructure of hypereutectic Al-Si alloys consisted of coarse primary silicon, slender eutectic silicon and α-Al. The size and morphology of primary silicon, which related to the Si content, had a significant effect on the mechanical properties of the hypereutectic Al-Si alloy. Compared with the traditional casting process of hypereutectic Al-Si alloys, the macroscopic segregation and coarse structure in 50 wt.% Si_p_/Al MMCs produced by the spray casting technique disappeared completely [24,25].

**Figure 2:**
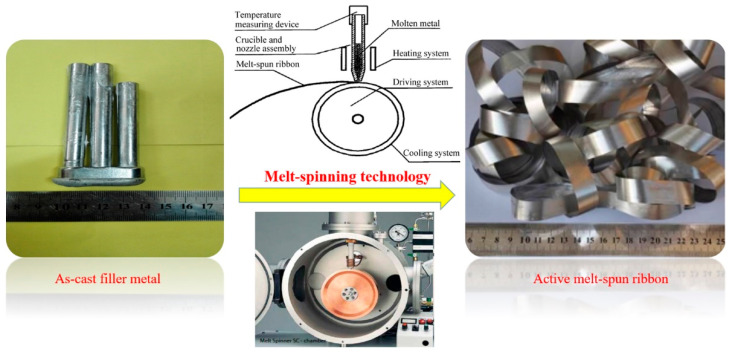
Schematic of the melt-spun ribbon preparation process.

The melt spinning process is the most common technique for manufacturing ultrafine-grained materials and even nano materials by freezing the molten metal at quite a rapid cooling rate with a water–cold copper disc. The melt spinning technology can produce microstructures which can vary greatly from those formed in traditional slow-cooled bulk material. Depending upon the alloy composition and cooling capacity, the cooling rates of molten metal ranged between 10^5^ to 10^8^ K/sec for this technology. When the cooling rates of liquid metal reached these values, non-equilibrium, metastable, micro-nano and even amorphous microstructures were possible [26]. A detailed schematic of the filler metal ribbon preparation is shown in Figure 2. After considerable experimentation and optimization, the composition of the investigated active brazing filler metal was designed based on the ternary eutectic Al-Si-Cu alloy, with the addition of Ni and Ti. Ni improved the formability of the melt-spun ribbon and reduced the intermetallic compounds such as CuAl_2_. The addition of Ti in the ribbon was done to refine the eutectic structures and enhance the interaction between the brazing filler metal and 50 wt.% Si_p_/Al MMCs. To prepare the active melt-spun ribbon, the as-cast master alloy Al-Si-Cu-Ni-Ti was smelted in an argon-shielded induction furnace and cast in an iron mold of 15 cm^3^ volume for three times, initially. Secondly, melt-spun ribbons were obtained by melt spinning technology through a nozzle with a size of 10 mm × 0.5 mm in a quartz crucible on a rapid rotating copper disc with a diameter of 28.0 cm, in a shielding argon atmosphere so as to avoid ribbon oxidation. The melt-spun process parameters can be controlled via the following equation to meet the requirement of ribbon thickness [27,28].

(1) $$d=\frac{2}{3}{\left(\frac{l}{d}\right)}^{\frac{1}{4}}\frac{b}{v}{\left(\frac{2p}{\rho }\right)}^{\frac{1}{2}}$$

where *d* is the ribbon thickness, *l* the distance between the nozzle and the surface of the copper disc, *b* the nozzle width, *v* the linear velocity of the copper disc, *p* the argon pressure at the nozzle, and *ρ* the alloy density under molten conditions. In this research, the ribbons were cast at a rotational speed of 1400 revolutions per minute, which equaled a linear velocity of 20.5 m/s, approximately.

Before vacuum brazing, the microstructural characteristics of the as-cast brazing filler metal and corresponding melt-spun ribbon were investigated. In order to determine the joining temperature, the thermodynamic property of the ribbon was measured by differential scanning calorimetry (DSC) at a heating rate of 10 °C/min. Brazing specimens with a size of 2.0 × 10.0 × 20.0 mm were machined from bulk materials of 50 wt.% Si_p_/Al MMCs and Kovar with an approximate dimension of 2.0 × 200.0 × 400.0 mm. Subsequently, the machined 50 wt.% Si_p_/Al MMCs and Kovar were mechanically polished on the bonding side with SiC grinding paper from 400# to 1200#, followed by ultrasonic cleaning in anhydrous ethanol for 5 min and drying in air. After preparation, the assembled specimens with brazing filler metal were joined in a vacuum furnace (ZHS-60, China Electronics Technology Group Corporation, Taiyuan, China) and then cooled to room temperature in the vacuum furnace before being taken out. During the joining and cooling process, the vacuum atmosphere was preset to better than 2 × 10^−3^ Pa to avoid material oxidation. After the joining process, the joint shear strength was tested by an electronic universal testing machine (CMT5205, MTS Systems (China) Co., Ltd., Shenzhen, China) with a constant shear rate of 0.02 mm/min at room temperature. For each of joining conditions, five samples were taken for the shearing test to obtain the average shearing data. An observation of the joined seam was carried out to study the microstructure and composition of the joint as well as the fracture surfaces by using a scanning electron microscope (Carl Zeiss NTS GmbH, Merlin Compact, Jena, Germany), secondary electron imaging (SEM) and energy-dispersive X-ray spectroscopy (EDS). The phase composition of the joint was determined using a D8 ADVANCE X-ray diffractometer (Bruker, Karlsruhe, Germany). The joint gas tightness was tested with a ZQJ-530 helium leak mass spectrometer (KYKY Technology Development Ltd., Beijing, China).

## 3. Results and Discussion

### 3.1. Microstructural Characteristics and Thermodynamic Property of Brazing Filler Metal

**Figure 3:**
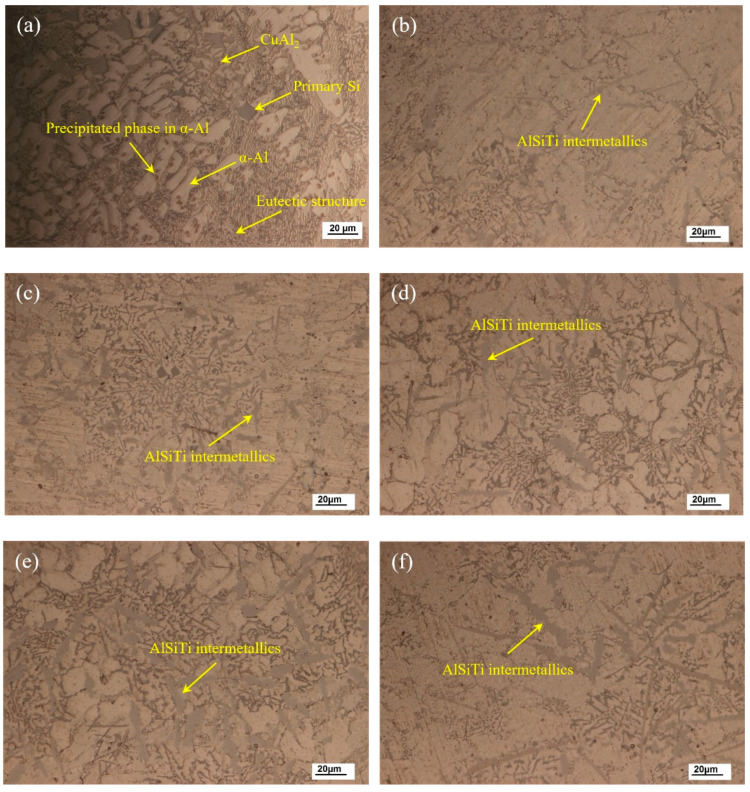
Optical micrographs of as-cast Al-7.5Si-23.0Cu-2.0Ni-xTi alloys: (**a**) Al-7.5Si-23.0Cu-2.0Ni-0.0Ti; (**b**) Al-7.5Si-23.0Cu-2.0Ni-0.5Ti; (**c**) Al-7.5Si-23.0Cu-2.0Ni-1.0Ti; (**d**) Al-7.5Si-23.0Cu-2.0Ni-1.5Ti; (**e**) Al-7.5Si-23.0Cu-2.0Ni-2.0Ti; (**f**) Al-7.5Si-23.0Cu-2.0Ni-2.5Ti.

Figure 3 shows the optical micrograph of the as-cast microstructures of brazing filler metal Al-7.5Si-23.0Cu-2.0Ni with Ti additions of 0.0, 0.5, 1.0, 1.5, 2.0, and 2.5 wt.%, respectively. As shown in Figure 3a, primary α-Al dendrites and a eutectic structure were the main phases in the as-cast Al-7.5Si-23.0Cu-2.0Ni microstructure. Meanwhile, the intermetallic compound CuAl_2_, a few primary Si, and the precipitated phase in α-Al were observed to coexist in the as-cast Al-7.5Si-23.0Cu-2.0Ni alloy as well. It can be seen that, in Figure 3b–f, the microstructure of the as-cast Al-7.5Si-23.0Cu-2.0Ni-xTi alloy changed significantly with the addition of Ti from 0.5 to 2.5 wt.% in comparison with Al-7.5Si-23.0Cu-2.0Ni. Firstly, the amount of primary Si and the eutectic structure were reduced, which mainly resulted from the Si consumption in the alloy due to the formation of intermetallic compounds containing Si elements. Secondly, new AlSiTi intermetallics were observed in Ti-containing filler metal, which mainly stem from the high chemical activity of Ti tending to react with Al and Si in alloys to form a stable and metastable phase, such as AlTi_2_, AlTi_3_ and Ti_7_Al_5_Si_12_, etc. It is reported that Ti-based intermetallics can have three different morphologies (flakes, petals and blocks), which mainly depend on the temperature history and solidification conditions of the alloys [29]. As can be seen in Figure 3b–f, the flake-like AlSiTi intermetallic phases increased and became slightly coarsened with the Ti addition from 0.5 to 2.5 wt.%.

**Figure 4:**
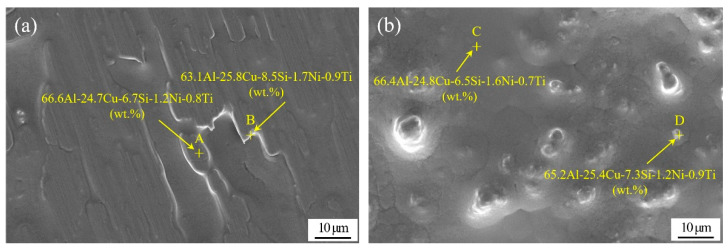
SEM image and EDS analysis of melt-spun ribbon of Al-7.5Si-23.0Cu-2.0Ni-1.0Ti taken from two sides: (**a**) contact surface; (**b**) free surface.

By melt-spun technology, a continuous Al-Si-Cu-Ni-Ti ribbon was successfully produced with a thickness of 90–140 μm and a width of 8 mm. The macroscopic observation demonstrated that the prepared ribbon had two different surface morphologies containing a smooth surface and a rough surface. Figure 4 shows the SEM and EDS analysis of the melt-spun ribbon with the nominal chemical composition of Al-7.5Si-23.0Cu-2.0Ni-1.0Ti taken from the contact surface and free surface, corresponding to a smooth surface and a rough surface, respectively. As can be seen, the melt-spun ribbon obtained a fine, tight and uniform microstructure on both sides. No holes and porosity existed in the ribbon. EDS analysis in Figure 4 also exhibited that the chemical compositions on both sides of the ribbon were very close to the nominal composition of the matrix alloy, which suggested that the distribution of the chemical composition in the ribbon was extremely homogeneous. Based on previous research, the grain size of our melt-spun ribbon on two sides was different because of the different cooling rate. Due to the maximum supercooling rate, the material at the contact surface exhibited noncrystalline characteristics, such as a low melting point, good shaping ability, high solid solubility and diffusion coefficient. The grain size on the free surface was much bigger than that of the contact surface due to the decrease in the supercooling rate along the thickness direction. In general, the grain size of the near-eutectic Al-Si-Cu-based active alloy manufactured by the melt-spun technique was around 200 nm due to the great supercooling rate [30]. Essentially, the solidification rate plays a key role in solidified structure and solute segregation, and the interrelation can be found in Equation (2). Equation (2) demonstrates that the critical grain radius ($r_{k}$) is inversely proportional to the supercooling rate (ΔT), suggesting an $r_{k}$ decrease with the increase in ΔT. During vacuum brazing, the grains in the brazing filler metal, characterized by their small crystal size and lesser regional segregation, will melt at a lower temperature, which can improve the wettability and spreadability of the brazing filler metal significantly. Therefore, the quality of the brazed joint will be improved.

(2) $$r_{k}=\frac{2\sigma T_{m}}{L_{m}\Delta T}$$

where $r_{k}$ is the critical grain radius, σ the surface energy for the crystal nucleus, $T_{m}$ the crystallization temperature in theory, $L_{m}$ the latent heat of fusion, and ΔT the supercooling rate.

**Figure 5:**
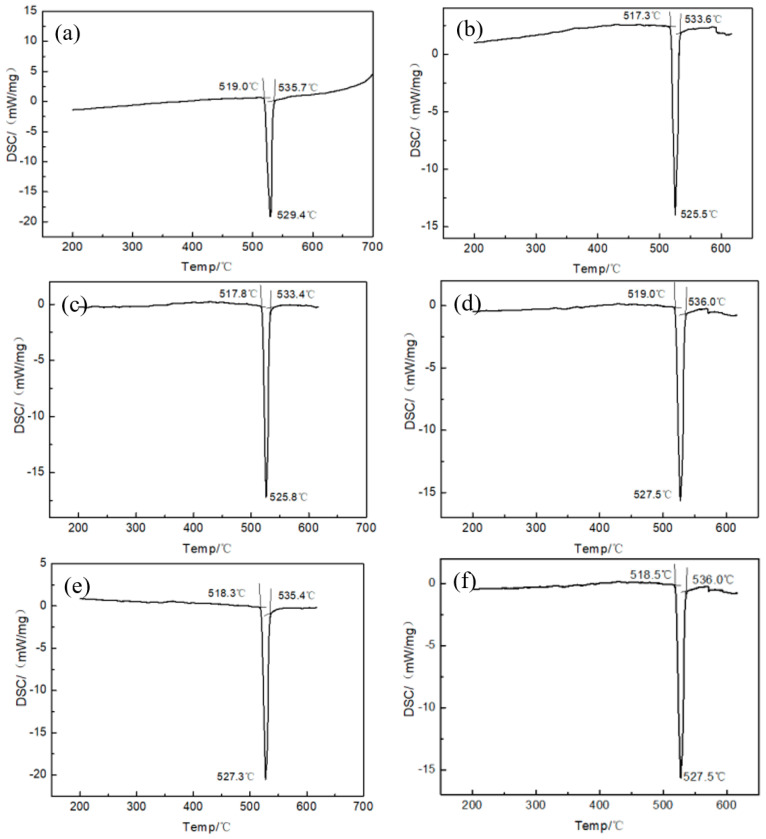
Differential scanning calorimetry (DSC) curve of the melt-spun ribbons with chemical compositions of (**a**) Al-7.5Si-23.0Cu-2.0Ni-0.0Ti; (**b**) Al-7.5Si-23.0Cu-2.0Ni-0.5Ti; (**c**) Al-7.5Si-23.0Cu-2.0Ni-1.0Ti; (**d**) Al-7.5Si-23.0Cu-2.0Ni-1.5Ti; (**e**) Al-7.5Si-23.0Cu-2.0Ni-2.0Ti; (**f**) Al-7.5Si-23.0Cu-2.0Ni-2.5Ti.

Figure 5 reveals the DSC curve of the melt-spun ribbon with a Ti addition from 0.0 to 2.5 wt.% in the Al-7.5Si-23.0Cu-2.0Ni alloy. The solidus and liquidus temperature of melt-spun ribbons with different chemical compositions are summarized in Table 2. It can be seen that the ribbons with a chemical composition of Al-7.5Si-23.0Cu-2.0Ni-xTi have a melting range from 517.3 °C to 536.0 °C. Meanwhile, the ribbon shows only one endothermic peak with a melting temperature, suggesting that it is close to the Al-Si-Cu eutectic alloy. As shown in Figure 5c, the ribbon with the composition of Al-7.5Si-23.0Cu-2.0Ni-1.0Ti exhibits the minimum melting range of 15.6 °C in compassion with the other compositions. Compared with the Al-5.1Si-26.7Cu ternary eutectic alloy characterized by a eutectic temperature of 525 °C, the solidus of the produced melt-spun ribbon, Al-7.5Si-23.0Cu-2.0Ni-xTi, decreased around 7 °C due to the formation of micro-nano-sized grains during the preparation. Micro-nano-sized grains with a diameter of 200 nm have high surface energy, giving them a lower solidus temperature compared with the bulk materials. Meanwhile, this high surface energy will be the main driving force of atomic motion, which will facilitate the diffusion into the matrix. In addition to this, the corresponding liquidus of the ribbon increased by 10 °C, approximately, due to the addition of high-temperature elements such as Ni and Ti.

### 3.2. Microstructure and Element Distribution Analysis of Brazed Joint

**Figure 6:**
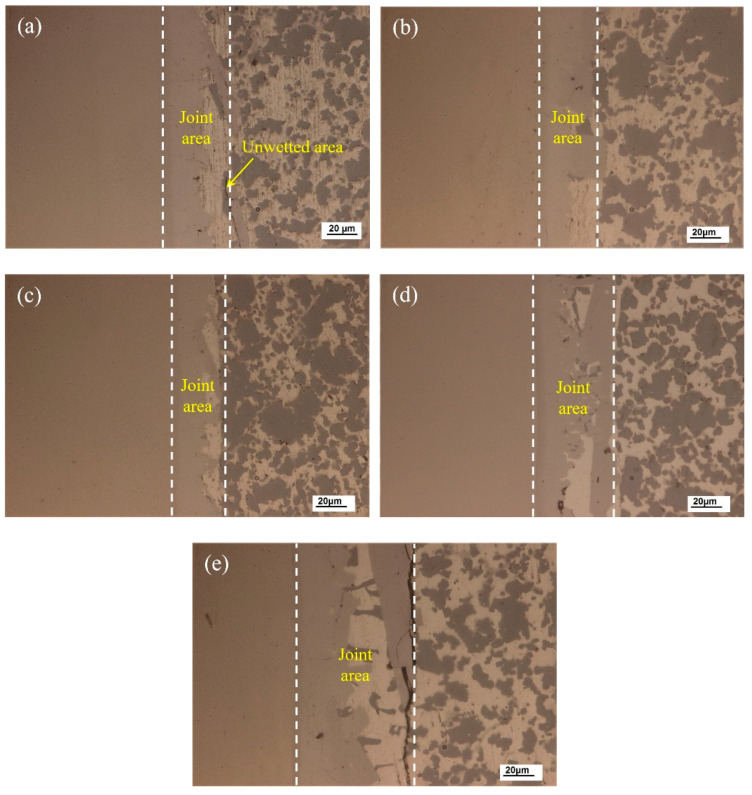
Optical micrographs of the joint made using different brazing temperatures: (**a**) 560 °C; (**b**) 570 °C; (**c**) 580 °C; (**d**) 590 °C; (**e**) 600 °C.

The joining temperature significantly affects the joint microstructure and properties. Based on the solidus and liquidus of the developed brazing filler metal, joining temperatures of 560, 570, 580, 590 and 600 °C were applied to the vacuum brazing of 50 wt.% Si_p_/Al MMCs to Kovar alloys in this research. Figure 6 shows the joint optical microstructure evolution at different joining temperatures using Al-7.5Si-23.0Cu-2.0Ni-1.0Ti as the filler metal for a brazing time of 30 min. The wettability and interaction between the filler metal and substrate was quite weak at lower joining temperatures due to the lower atomic activity. As shown in Figure 6a, a small defect such as an unwetted area exists at the interface between the filler metal and 50 wt.% Si_p_/Al MMCs, which may influence the joint shear strength and gas tightness. In Figure 6b, a small part of the Si particles dissolve into the brazing seam during the process. It can be clearly seen in Figure 6c that the brazed joint is continuous and tightly jointed, without defects such as pores and cracks, with a joining temperature of 580 °C. Good physical and chemical metallurgical bonding of the filler metal to the substrate was obtained. Melt-spun ribbons characterized by micro-nano grains have high surface energy, which is the main driving force of atomic motion that facilitates the diffusion into Kovar and 50 wt.% Si_p_/Al MMCs. In Figure 6d,e, more intermetallic phases, primarily including AlSiTi, are generated in the joint due to the higher joining temperature. When the joining temperature reaches 600 °C, some micro-cracks appear at the interface between the filler metal and the 50 wt.% Si_p_/Al MMCs. As a consequence, the joint properties, such as shearing strength and gas tightness, will be undesirable, which will be verified in the following sections.

**Figure 7:**
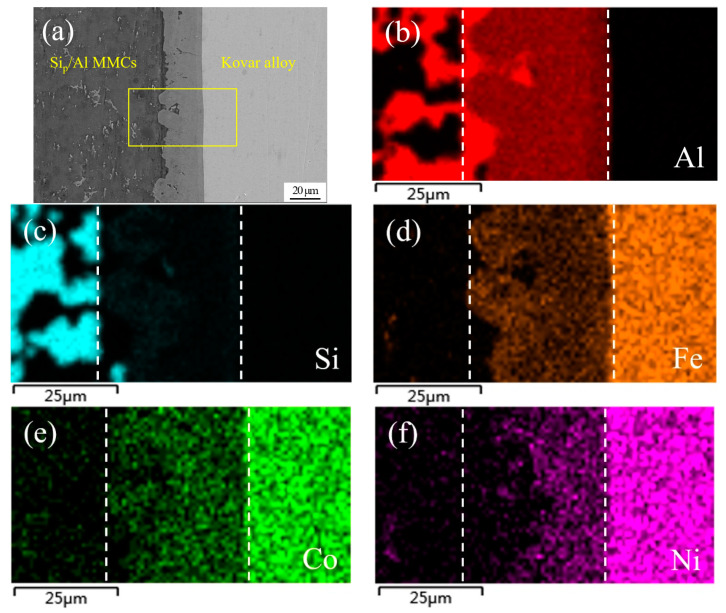

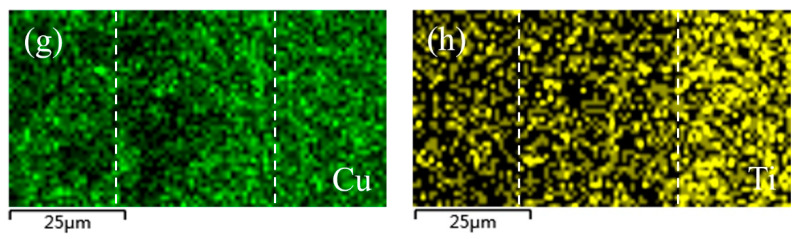
SEM image of typical region at the interface of 50 wt.% Si_p_/Al MMCs/Kovar made using Al-7.5Si-23.0Cu-2.0Ni-1.0Ti at 580 °C for 30 min and corresponding energy dispersive X-ray maps showing distribution of elements: (**a**) SEM image; (**b**–**h**) individual elemental mapping of Al, Si, Fe, Co, Ni, Cu, and Ti, respectively.

Figure 7 shows an SEM image of a typical region in the joint made using Al-7.5Si-23.0Cu-2.0Ni-1.0Ti as the brazing filler metal at 580 °C for 30 min and the corresponding elemental mapping. As can be seen in Figure 7a, the interface between the brazing filler metal and the 50 wt.% Si_p_/Al MMCs was not very smooth, which primary resulted from the strong interaction between the filler metal and the 50 wt.% Si_p_/Al MMCs. Meanwhile, plenty of phase boundaries in the 50 wt.% Si_p_/Al MMCs were quite beneficial to the element diffusion, which can accelerate the element interaction between the ribbon and the 50 wt.% Si_p_/Al MMCs. On the other hand, the interface between the brazing filler metal and the Kovar alloys was quite flat, the main reason being that Kovar alloys consist of only single-phase austenite during the brazing condition. Moreover, the phase boundary, which could be very useful for the interaction, cannot be found in Kovar alloys. As a consequence, the rate of interaction between the ribbon and the Kovar alloys was much lower than that in the 50 wt.% Si_p_/Al MMCs. As shown in Figure 7b–h, the compositions in the brazing seam included all chemical elements in the filler metal and base metals due to the interaction under the joining condition. The brazing seam is identified by two dashed lines in Figure 7. Obviously, the elements in the Kovar alloys, including Fe, Co and Ni, diffused into the brazing seam in a large quantity, as shown in Figure 7d–f. Furthermore, a small amount of Co and Ni was detected in 50 wt.% Si_p_/Al MMCs near the brazing seam. Al was the matrix element in the brazing seam. However, the distribution of Al in the brazing seam was not uniform, as presented in Figure 7b. Al segregation appeared in the brazing area near the 50 wt.% Si_p_/Al MMCs, which resulted from the dissolution of Al from the 50 wt.% Si_p_/Al MMCs. In addition, it was hard to find Al on the Kovar side. It was hard to detect Si in most areas of the brazing seam, except some small areas close to the 50 wt.% Si_p_/Al MMCs, just like in the bottom left of the brazing seam in Figure 7c. The main reason was that Si was ready to crystallize on the base of existing Si particles in the Si_p_/Al MMCs during the solidification process. Similarly, it was hard to find Si on the Kovar side. Figure 7g,h shows that the distribution of Cu and Ti was quite homogenous in both the base metals and the brazing seam, since Cu and Ti had large diffusion coefficients in those materials. Meanwhile, Cu and Ti showed great solid solubility and high activity in those materials, respectively.

**Figure 8:**
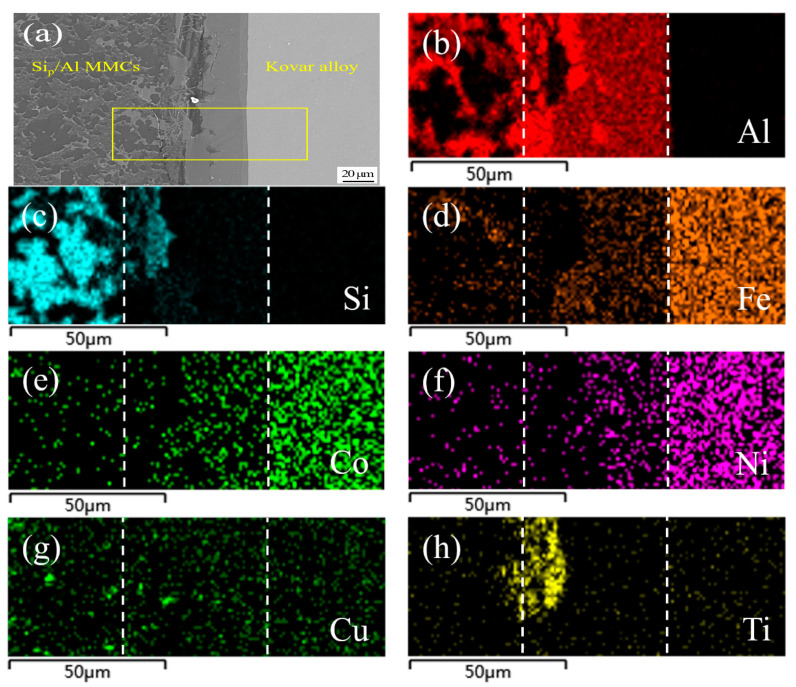
SEM images of typical region at the interface of 50 wt.% Si_p_/Al MMCs/Kovar made using Al-7.5Si-23.0Cu-2.0Ni-2.5Ti at 580 °C for 30 min and corresponding energy dispersive X-ray maps showing distribution of elements: (**a**) SEM image; (**b**–**h**) individual elemental mapping of Al, Si, Fe, Co, Ni, Cu, and Ti, respectively.

Figure 8 shows SEM images and the corresponding elemental mapping of a typical region in the joint made using Al-7.5Si-23.0Cu-2.0Ni-2.5Ti as the filler metal and the same process parameters as in Figure 7. Obviously, the higher content of Ti in the filler metal resulted in a different joint microstructure and properties. As presented in Figure 8a, massive flakes appeared at the interface between the brazing seam and 50 wt.% Si_p_/Al MMCs, and a small amount of the flakes even appeared in the 50 wt.% Si_p_/Al MMCs close to the interface. As shown in Figure 8b,c,h, elemental mapping analysis showed that Al, Si and Ti were the main compositions in that flake area. Clearly, the addition of Ti had significant control over the formation of AlSiTi intermetallic phases. Generally, the appearance of a massive intermetallic phase in the joint interface was not good for the mechanical properties and gas tightness of the joints due to their brittleness, and this is confirmed in the following section.

**Figure 9:**
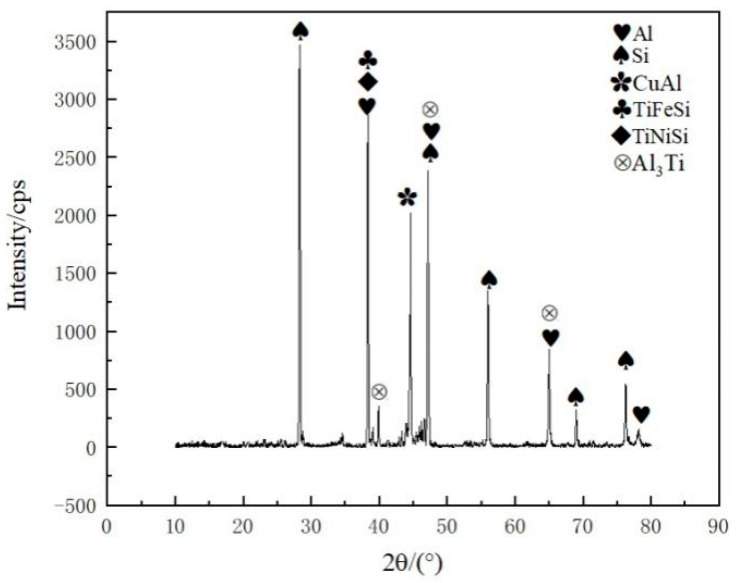
XRD analysis of vacuum brazing joint made using Al-7.5Si-23.0Cu-2.0Ni-1.0Ti as filler metal at brazing temperature of 580 °C and brazing time of 30 min.

An XRD analysis of the joint is presented in Figure 9 and reveals peaks for several intermetallic compounds when the filler metal Al-7.5Si-23.0Cu-2.0Ni-1.0Ti, at a brazing temperature of 580 °C and a brazing time of 30 min, was applied. The main phases in the joint were mainly composed of Al, Si and intermetallics, including CuAl, TiFeSi, TiNiSi and Al_3_Ti. It was found that the elements in the Kovar alloys, such as Fe and Ni, were dissolved into the brazing filler metal and an interaction occurred, forming the intermetallics TiFeSi and TiNiSi. Combined with the elemental mapping in Figure 7, XRD analysis suggested that those small intermetallics were distributed in the Al solid solution quite homogeneously in order to reinforce the brazing seam.

### 3.3. Mechanical Property and Gas Tightness of Brazed Joint

**Figure 10:**
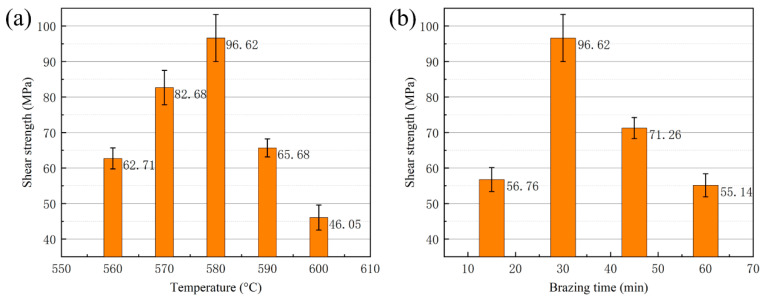
Shear strength of brazed joint as a function of (**a**) brazing temperature with constant brazing time of 30 min; (**b**) brazing time with constant brazing temperature 580 °C.

The shear strength of a vacuum-brazed joint has a significant influence on its practical applications. Figure 10 shows the room temperature shear strength of the brazed joint at different brazing temperatures and brazing times when using Al-7.5Si-23.0Cu-2.0Ni-1.0Ti as the filler metal. In Figure 10a,b, a brazing time of 30 min and brazing temperature of 580 °C were utilized, respectively. As shown in Figure 10a, the shear strength increased with the increasing temperature from 560 °C to 580 °C. The maximum shear strength of the brazed joint was 96.62 MPa at a brazing temperature of 580 °C for 30 min. Once the temperature exceeded 580 °C, the joint shear strength decreased significantly. The minimum shear strength of the brazed joint was 46.05 MPa when a brazing temperature of 600 °C was applied. With an increase in the joining temperature, the diffusion coefficient of elements in the filler metal increased significantly. Meanwhile, a higher diffusion coefficient in the elements would lead to a change in the quantity and morphology of the intermetallic compounds in the weld seam, which have a remarkable impact on joint shear strength. In addition, a few liquid aluminums effused from the 50 wt.% Si_p_/Al MMCs during the brazing process when a joining temperature of 600 °C was applied. This phenomenon degraded the joint shear strength and matrix property greatly. The brazing time has a similar influence on joint shear strength, as displayed in Figure 10b. The maximum shear strength was obtained when a brazing time of 30 min was applied. As described in Equation (3), the diffusion flux in the joint is a function of the brazing temperature.

(3) $$d_{m}=-DS\frac{d_{c}}{d_{x}}d_{t},\;\;\;x^{2}=D\left(t-t_{0}\right)$$

where $d_{m}$ is the diffusion flux, D the diffusion coefficient, S the contact area between the filler metal and the matrix, x the diffusion distance, *d_c_/d_x_* the chemical concentration gradient, t the brazing time, and $t_{0}$ the latent time before diffusion.

It can be seen in Equation (3) that the brazing time has an effect on the diffusion flux and the diffusion distance of the filler metal. If the brazing time was prolonged appropriately, a considerable metallurgical reaction along the joining interface could occur. From the test of shear strength, a brazing time of 30 min was approved to be optimal in this research. When the brazing time was reduced to 15 min, the joint shear strength decreased to 56.76 MPa due to the insufficient diffusion between the brazing filler metal and the matrix. Instead, if the brazing time exceeded 30 min, the joint shear strength decreased as well, since more brittle intermetallics could be formed during the process.

**Figure 11:**
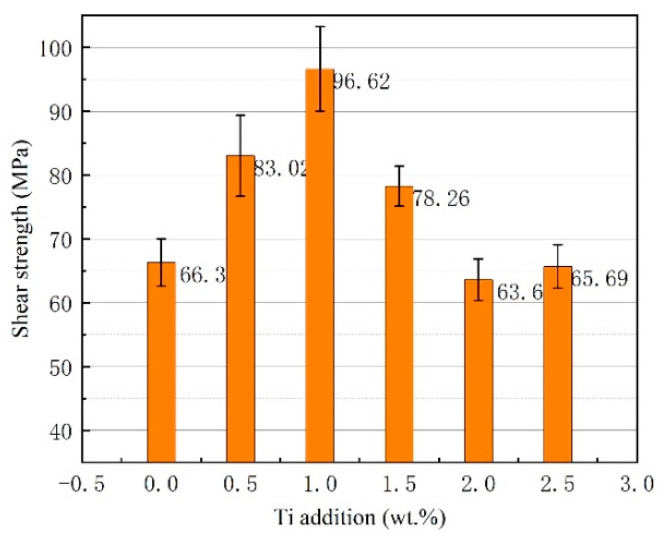
Shear strength of brazed joint as a function of Ti addition under the brazing temperature of 580 °C and brazing time of 30 min.

Figure 11 shows the room temperature shear strength of the brazed joint as a function of the Ti addition under a brazing temperature of 580 °C and a brazing time of 30 min. It can be seen that Ti addition in brazing filler metal has a significant influence on joint shear strength. As the Ti addition was under 1.0 wt.%, the joint shear strength increased gradually. The increase in shear strength could be attributed to the effective blocking of the dislocation motion after the addition of Ti, forming a few of the intermetallics in the joint, and this was confirmed in a previous XRD analysis of the joint. Once the Ti addition exceeded 1.0 wt.%, the joint shear strength decreased quickly because of the formation of massive brittle intermetallic phases due to the occurrence of an excessive metallurgical reaction in joint, as presented in Figure 8a.

One of the important application domains of 50 wt.% Si_p_/Al MMC/Kovar is electronic packaging, which requires a very strict joint gas tightness to protect the inside chip from potential damage caused by surrounding air during service. After the brazing process, the joining area is the only potential leakage path if joining defects exist, such as micro-cracks, gaps, porosity, and a lack of bonding, etc. To measure the gas tightness of the joint, a specimen with a hole in the center of the 50 wt.% Si_p_/Al MMCs was produced and then bonded with the Kovar alloy. As a qualified component used in the electronic packaging field, the gas leakage rate after the bonding process is supposed to be maintained at 10^−8^ Pa·m^3^/s or below. After the joining process, the gas tightness of the specimens was tested by utilizing the ZQJ-530 helium leak mass spectrometer. The tested results are displayed in Table 3.

It can be seen in Table 3 that the brazed joints meet the gas tightness requirement for electronic packaging devices by using brazing temperatures of 570 °C, 580 °C and 590 °C with a brazing time of 30 min. When a brazing temperature of 560 °C was applied, the joint leakage rate after vacuum brazing was 10^−7^ Pa·m^3^/s, which was too high and failed to meet the requirement for packaging devices. Lower joining temperatures of 560 °C may cause incomplete joining at the interface, reducing the compactness of the weld seam due to micro-voids inevitably being left in the weld seam. However, the joint leakage rate was reduced to 10^−6^ Pa·m^3^/s when a brazing temperature of 600 °C was applied. The degradation of the substrate took place, since a few liquid aluminums effused from the 50 wt.% Si_p_/Al MMCs under a brazing temperature of 600 °C. The stability tests were carried out after one month. As shown in Table 3, the leakage rate of the specimens was unchanged, suggesting that the joint possessed good stability.

### 3.4. Fracture Analysis of Brazed Joint

**Figure 12:**
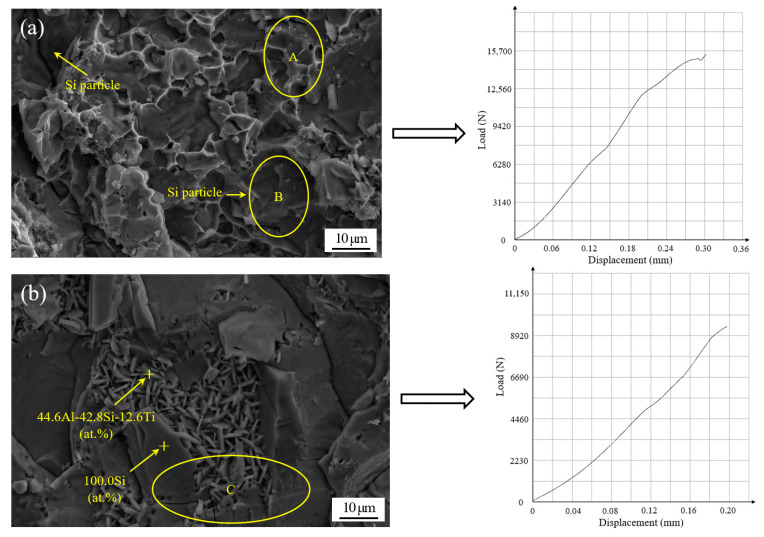
Scanning fracture appearance and corresponding shear curve of the joints brazed at temperature of 580 °C and time of 30 min with different brazing filler metals: (**a**) Al-7.5Si-23.0Cu-2.0Ni-1.0Ti; (**b**) Al-7.5Si-23.0Cu-2.0Ni-2.5Ti.

The different microstructures of the joints result in a diverse fracture morphology. Figure 12 displayed the typical fracture morphology and corresponding shear curve of the brazed joints under different brazing conditions after room temperature shear testing. In Figure 12, the joints were brazed with different filler metals and the same process parameters, including a brazing temperature of 580 °C and a brazing time of 30 min. As shown in Figure 12a, the fracture is generally in the form of a quasi-cleavage fracture. Some areas of fracture, such as ellipse A, display ductile fractures in the filler metal and the Al matrix alloy. Moreover, some other areas, such as ellipse B, display the brittle style found on the 50 wt.% Si_p_/Al MMC side, where Si particles can be seen clearly. As a consequence, the fracture primarily occurs at the interface between the brazing filler metal and the 50 wt.% Si_p_/Al MMCs. The fracture appearance also demonstrates that there are no large blocked intermetallic compounds in the joint, and this would be very beneficial in terms of increasing the joint strength. The corresponding shear curve indicates that some yield phenomena appear due to the ductile fractures in some areas. Figure 12b shows that a number of growing AlSiTi intermetallic phases appear at the fracture when the addition of active Ti in the filler metal increases to 2.5 wt.%. The corresponding shear curve also shows that the fracture mode is a typical brittle fracture. Using EDS analysis, the chemical composition of the flake-like phase was measured to be 44.6Al-42.8Si-12.6Ti at.%, which indicated that Ti(AlSi)_3_ was the primary intermetallic phase at the fracture surface. The average size of the Ti(AlSi)_3_ intermetallic phase is around 7 µm. Furthermore, this result was consistent with the analysis in Figure 8. A block of Si, surrounded by Ti(AlSi)_3_, can also be seen in Figure 12b. Meanwhile, a partial micro-crack appeared in Figure 12b, as marked in ellipse C. The micro-crack propagated not only the newly generated Ti(AlSi)_3_ phase, but also the Si particles in the substrate. During the brazing process, the production of a brittle Ti(AlSi)_3_ phase caused stress concentration region at the interface [29]. In the following shearing test, the joint was easily broken in this region of stress concentration. Meanwhile, some micro-cracks were left in the fracture due to the brittleness of the intermetallic phase and the Si particles.

## 4. Conclusions

In this work, dissimilar electronic packaging materials, 50 wt.% Si_p_/Al MMCs, and Kovar alloys were successfully joined through a brazing process in a vacuum environment. Active melt-spun ribbons with a chemical composition of Al-7.5Si-23.0Cu-2.0Ni-xTi were utilized as brazing filler metals at brazing temperatures of 560–600 °C, with a brazing time of 15–60 min. The following conclusions can be drawn:

(1) Using melt-spun technology, a continuous Al-7.5Si-23.0Cu-2.0Ni-xTi ribbon, which presented very good edge definition, was prepared successfully with a thickness of 90–140 µm and a width of 8 mm. The distribution of the chemical composition in the ribbon was extremely homogeneous. The ribbon with a composition of Al-7.5Si-23.0Cu-2.0Ni-1.0Ti exhibits the minimum melting range of 15.6 °C in comparison with the other compositions.
(2) When the joint was made at 580 °C for 30 min, the Ti addition in the brazing filler metal had significant effect on the phase in the joint. Using Al-7.5Si-23.0Cu-2.0Ni-1.0Ti as brazing filler metal, the main phases in the joint were composed of Al, Si and small intermetallics, including CuAl, TiFeSi, TiNiSi and Al_3_Ti. With higher Ti content in the brazing filler metal, such as Al-7.5Si-23.0Cu-2.0Ni-2.5Ti, the joint contained a large amount of AlSiTi intermetallic phases at the interface between the brazing seam and the 50 wt.% Si_p_/Al MMCs.
(3) Using the brazing filler metal Al-7.5Si-23.0Cu-2.0Ni-1.0Ti, the maximum joint shear strength of 96.62 MPa was achieved when the optimal brazing temperature of 580 °C was applied for 30 min. The Ti addition in the brazing filler metal had a significant influence on joint shear strength. When the joint was brazed in the temperature range from 570 to 590 °C and for 30 min with the filler metal, Al-7.5Si-23.0Cu-2.0Ni-1.0Ti, the leakage rate of the joints was 10^−8^ Pa·m^3^/s or better, which meets the requirement of gas tightness for electronic packaging devices.
(4) For the vacuum brazing of 50 wt.% Si_p_/Al MMCs to Kovar alloys, the joint fracture was generally in the form of a quasi-cleavage fracture, which primarily occurred at the interface between the filler metal and the 50 wt.% Si_p_/Al MMCs. A number of flake-like Ti(AlSi)_3_ intermetallic phases appeared at the fracture when the addition of active Ti in filler metal increased to 2.5 wt.%. The average size of the Ti(AlSi)_3_ intermetallic phase was around 7 µm.

## Figures and Tables

**Table 1:** Chemical composition of silicon particle-reinforced aluminum matrix composites (Si_p_/Al MMCs) (in wt.%).

Element	Si	Mg	Fe	Zn	Mn	Al
wt.%	50.0	0.27	1.65	0.91	1.36	Balance

**Table 2:** Solidus and liquidus temperature of melt-spun ribbons with different chemical composition.

Chemical Composition of Melt-Spun Ribbon (wt.%)	Solidus Temperature (°C)	Liquidus Temperature (°C)
Al-7.5Si-23.0Cu-2.0Ni-0.0Ti	519.0	535.7
Al-7.5Si-23.0Cu-2.0Ni-0.5Ti	517.3	533.6
Al-7.5Si-23.0Cu-2.0Ni-1.0Ti	517.8	533.4
Al-7.5Si-23.0Cu-2.0Ni-1.5Ti	519.0	536.0
Al-7.5Si-23.0Cu-2.0Ni-2.0Ti	518.3	535.4
Al-7.5Si-23.0Cu-2.0Ni-2.5Ti	518.5	536.0

**Table 3:** Gas tightness of joints brazed by Al-7.5Si-23.0Cu-2.0Ni-1.0Ti at different temperatures.

Temperature (°C)	560	570	580	590	600
Leak rate after vacuum brazing (Pa·m^3^/s)	10^−7^	10^−9^	10^−10^	10^−8^	10^−6^
Leak rate after one month (Pa·m^3^/s)	10^−7^	10^−9^	10^−10^	10^−8^	10^−6^
